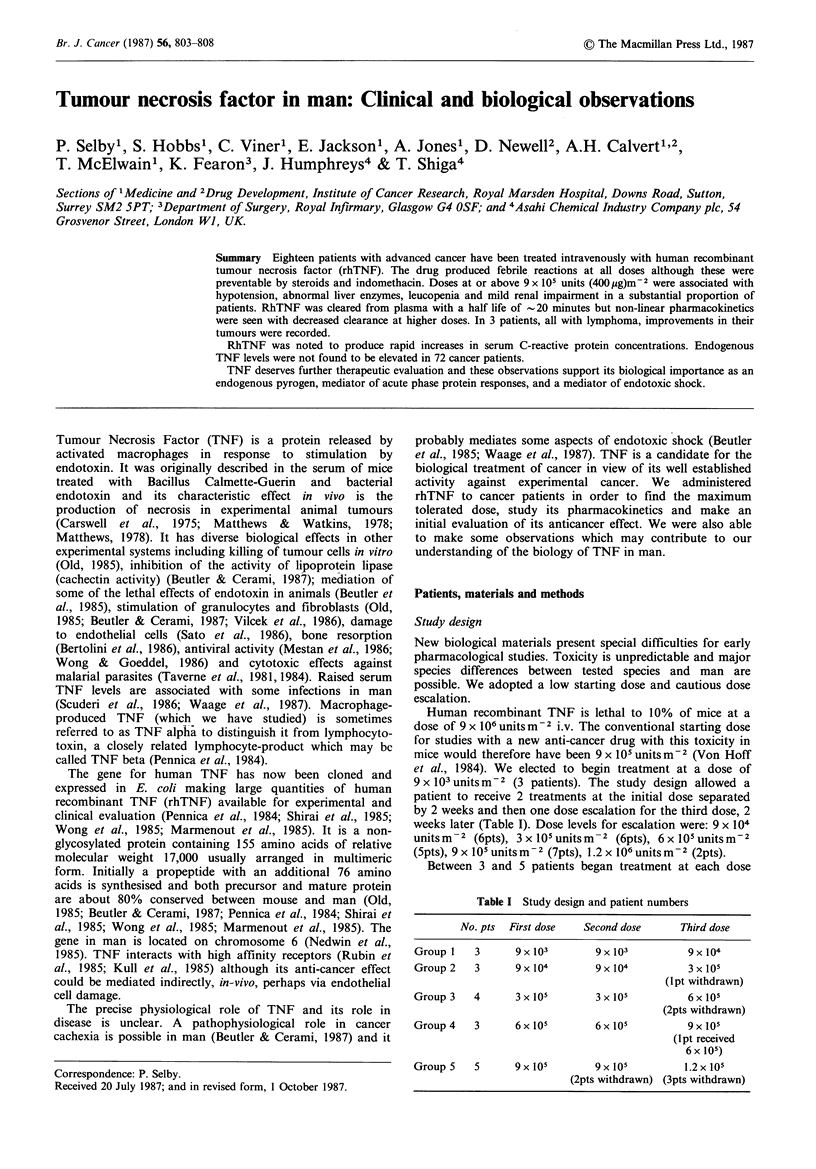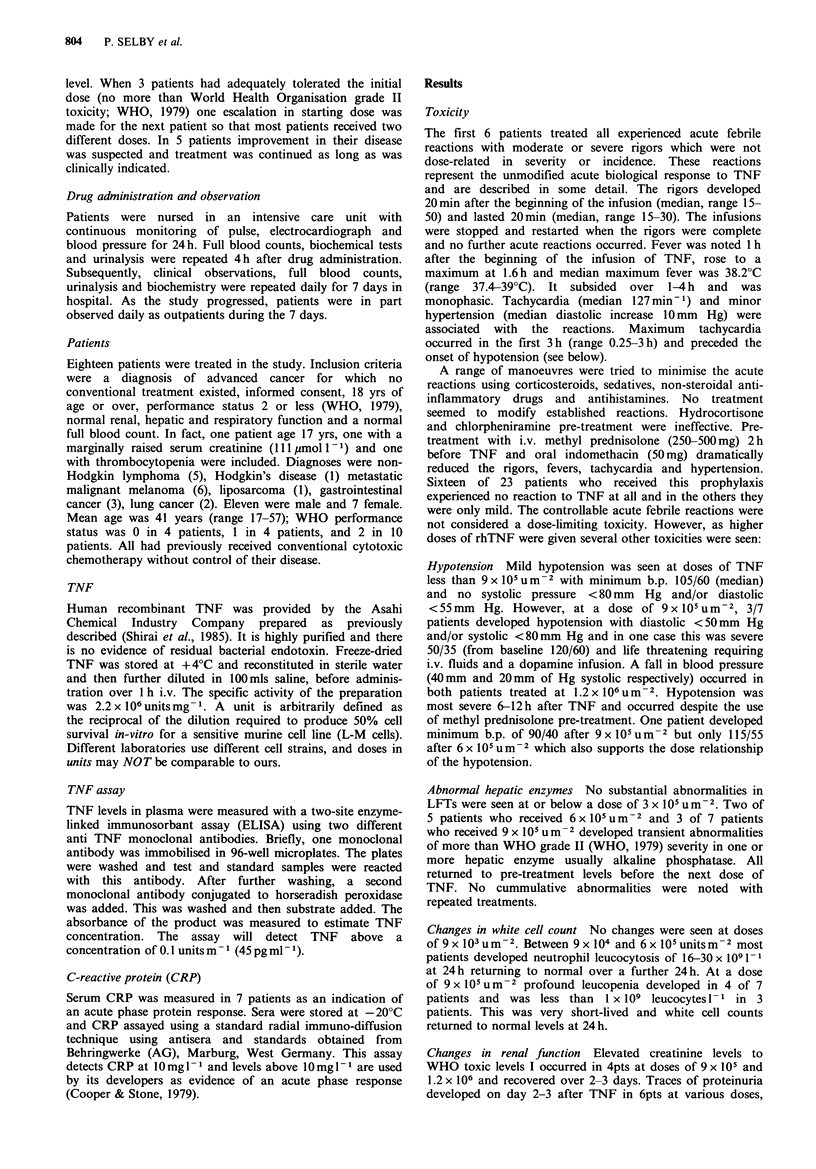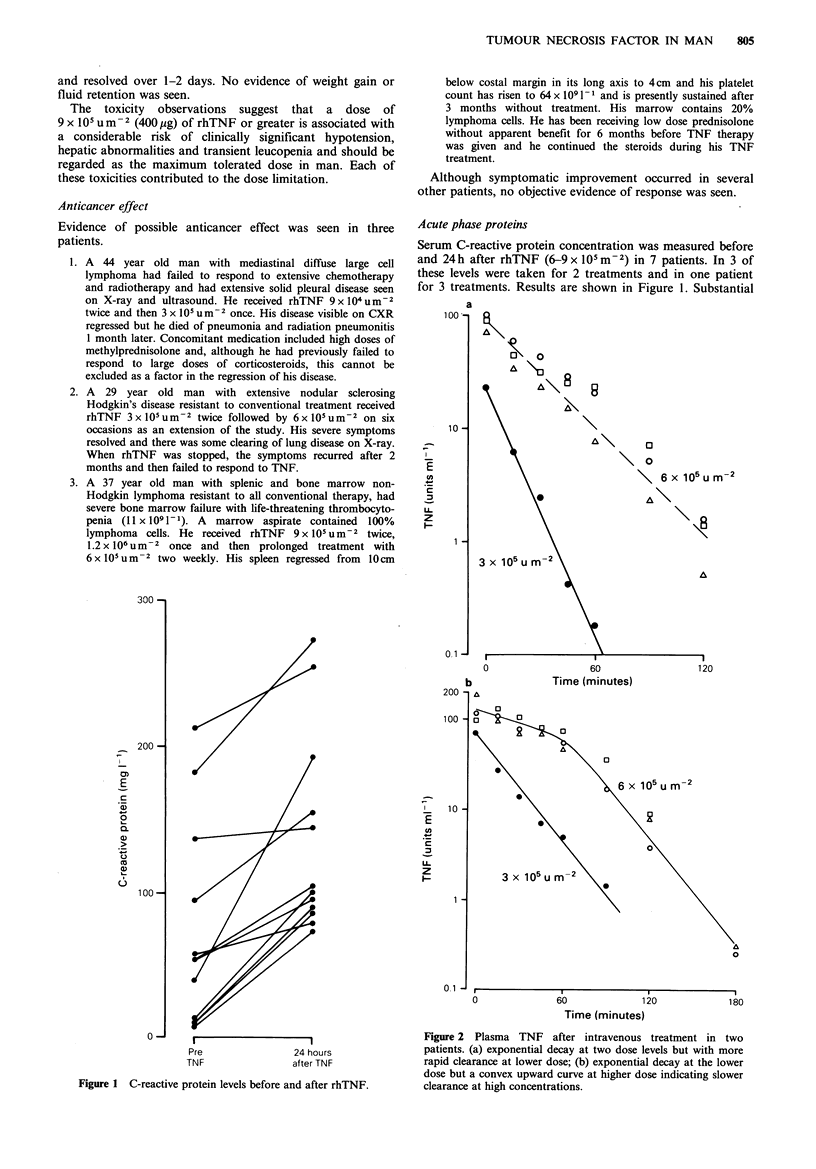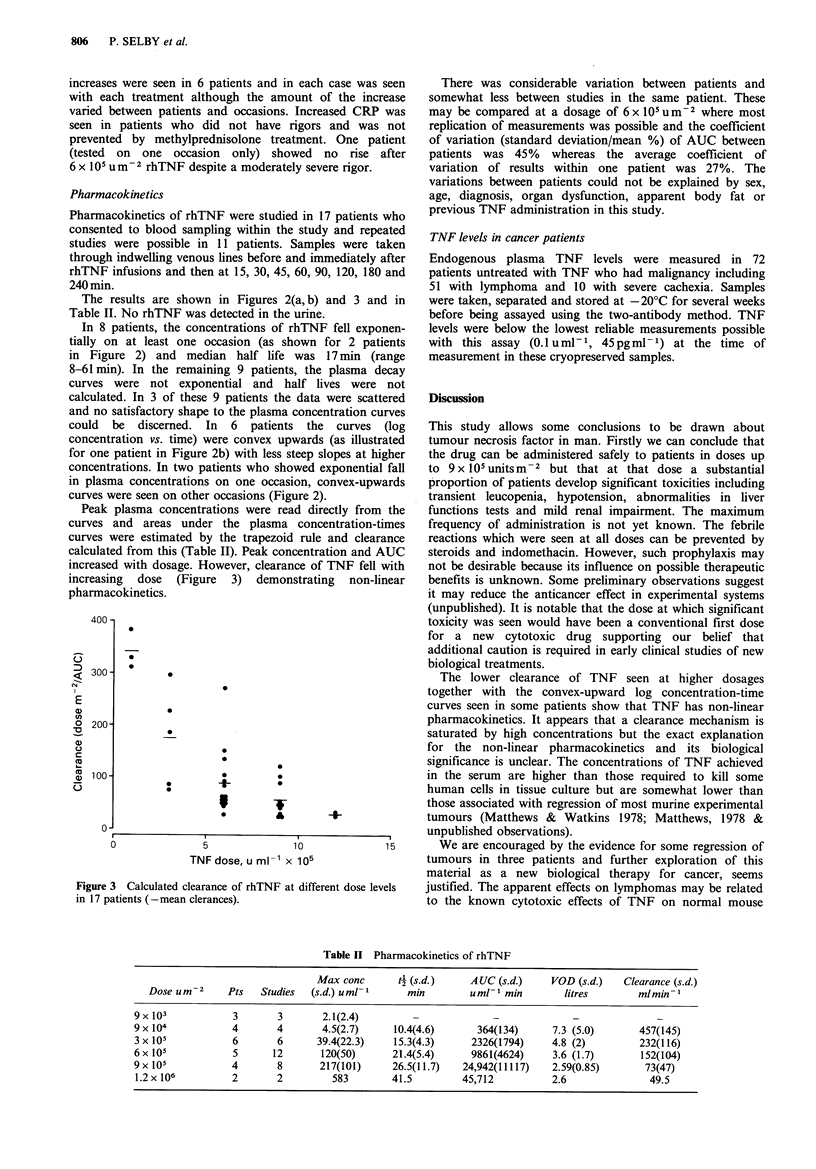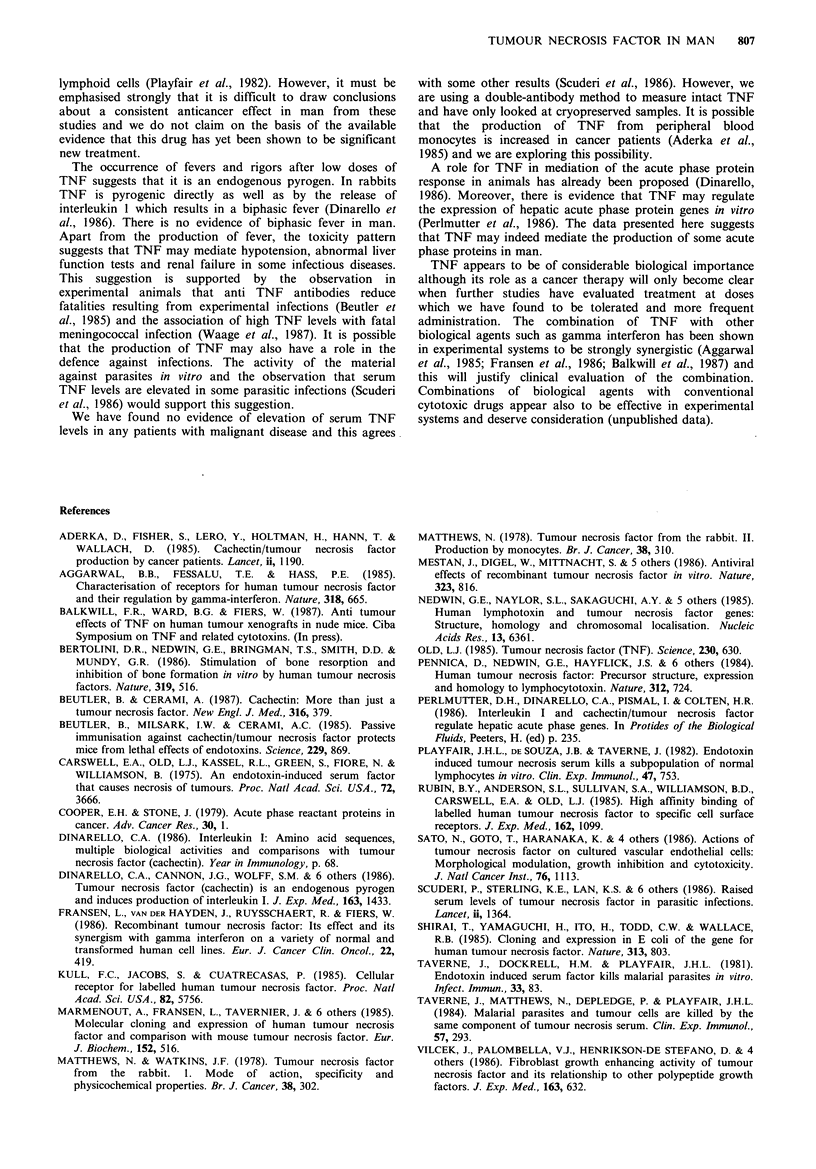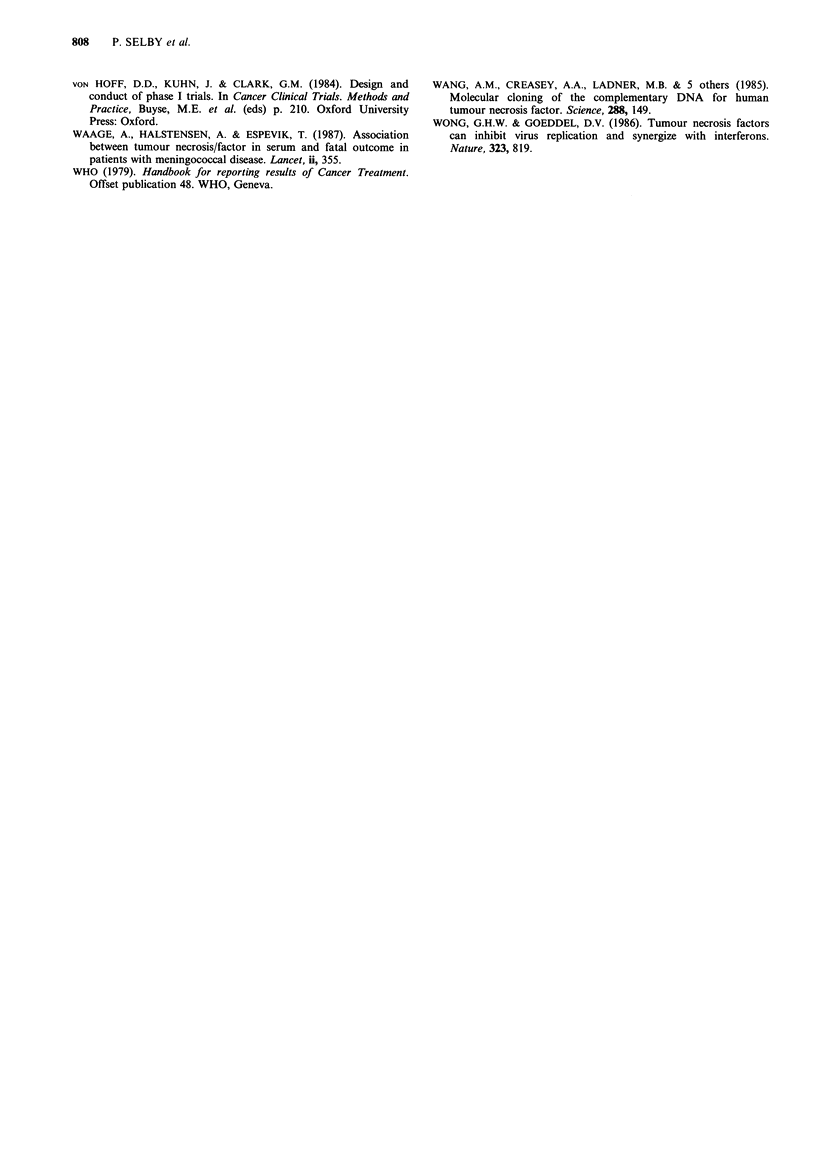# Tumour necrosis factor in man: clinical and biological observations.

**DOI:** 10.1038/bjc.1987.294

**Published:** 1987-12

**Authors:** P. Selby, S. Hobbs, C. Viner, E. Jackson, A. Jones, D. Newell, A. H. Calvert, T. McElwain, K. Fearon, J. Humphreys

**Affiliations:** Institute of Cancer Research, Royal Marsden Hospital, Sutton, Surrey, UK.

## Abstract

Eighteen patients with advanced cancer have been treated intravenously with human recombinant tumour necrosis factor (rhTNF). The drug produced febrile reactions at all doses although these were preventable by steroids and indomethacin. Doses at or above 9 x 10(5) units (400 micrograms)m-2 were associated with hypotension, abnormal liver enzymes, leucopenia and mild renal impairment in a substantial proportion of patients. RhTNF was cleared from plasma with a half life of approximately 20 minutes but non-linear pharmacokinetics lymphoma, improvements in their tumours were recorded. RhTNF was noted to produce rapid increases in serum C-reactive protein concentrations. Endogenous TNF levels were not found to be elevated in 72 cancer patients. TNF deserves further therapeutic evaluation and these observations support its biological importance as an endogenous pyrogen, mediator of acute phase protein responses, and a mediator of endotoxic shock.


*Br. J. Cancer* (1987) **56**, 803–808 © The Macmillan Press Ltd., 1987

### Tumour necrosis factor in man: Clinical and biological observations

P. Selby[1], S. Hobbs[1], C. Viner[1], E. Jackson[1], A. Jones[1], D. Newell[2], A.H. Calvert[1,2], T. McElwain[1], K. Fearon[3], J. Humphreys[4] & T. Shiga[4]

*Sections of [1]Medicine and [2]Drug Development, Institute of Cancer Research, Royal Marsden Hospital, Downs Road, Sutton, Surrey SM2 5PT; [3]Department of Surgery, Royal Infirmary, Glasgow G4 0SF; and [4]Asahi Chemical Industry Company plc, 54 Grosvenor Street, London W1, UK.*

**Summary** Eighteen patients with advanced cancer have been treated intravenously with human recombinant tumour necrosis factor (rhTNF). The drug produced febrile reactions at all doses although these were preventable by steroids and indomethacin. Doses at or above $9 \times 10^5$ units (400 $\mu$g)m$^{-2}$ were associated with hypotension, abnormal liver enzymes, leucopenia and mild renal impairment in a substantial proportion of patients. RhTNF was cleared from plasma with a half life of $\sim$20 minutes but non-linear pharmacokinetics were seen with decreased clearance at higher doses. In 3 patients, all with lymphoma, improvements in their tumours were recorded.

RhTNF was noted to produce rapid increases in serum C-reactive protein concentrations. Endogenous TNF levels were not found to be elevated in 72 cancer patients.

TNF deserves further therapeutic evaluation and these observations support its biological importance as an endogenous pyrogen, mediator of acute phase protein responses, and a mediator of endotoxic shock.

Tumour Necrosis Factor (TNF) is a protein released by activated macrophages in response to stimulation by endotoxin. It was originally described in the serum of mice treated with Bacillus Calmette-Guerin and bacterial endotoxin and its characteristic effect *in vivo* is the production of necrosis in experimental animal tumours (Carswell *et al.*, 1975; Matthews & Watkins, 1978; Matthews, 1978). It has diverse biological effects in other experimental systems including killing of tumour cells *in vitro* (Old, 1985), inhibition of the activity of lipoprotein lipase (cachectin activity) (Beutler & Cerami, 1987); mediation of some of the lethal effects of endotoxin in animals (Beutler *et al.*, 1985), stimulation of granulocytes and fibroblasts (Old, 1985; Beutler & Cerami, 1987; Vilcek *et al.*, 1986), damage to endothelial cells (Sato *et al.*, 1986), bone resorption (Bertolini *et al.*, 1986), antiviral activity (Mestan *et al.*, 1986; Wong & Goeddel, 1986) and cytotoxic effects against malarial parasites (Taverne *et al.*, 1981, 1984). Raised serum TNF levels are associated with some infections in man (Scuderi *et al.*, 1986; Waage *et al.*, 1987). Macrophage-produced TNF (which we have studied) is sometimes referred to as TNF alpha to distinguish it from lymphocytotoxin, a closely related lymphocyte-product which may be called TNF beta (Pennica *et al.*, 1984).

The gene for human TNF has now been cloned and expressed in *E. coli* making large quantities of human recombinant TNF (rhTNF) available for experimental and clinical evaluation (Pennica *et al.*, 1984; Shirai *et al.*, 1985; Wong *et al.*, 1985; Marmenout *et al.*, 1985). It is a non-glycosylated protein containing 155 amino acids of relative molecular weight 17,000 usually arranged in multimeric form. Initially a propeptide with an additional 76 amino acids is synthesised and both precursor and mature protein are about 80% conserved between mouse and man (Old, 1985; Beutler & Cerami, 1987; Pennica *et al.*, 1984; Shirai *et al.*, 1985; Wong *et al.*, 1985; Marmenout *et al.*, 1985). The gene in man is located on chromosome 6 (Nedwin *et al.*, 1985). TNF interacts with high affinity receptors (Rubin *et al.*, 1985; Kull *et al.*, 1985) although its anti-cancer effect could be mediated indirectly, *in-vivo*, perhaps via endothelial cell damage.

The precise physiological role of TNF and its role in disease is unclear. A pathophysiological role in cancer cachexia is possible in man (Beutler & Cerami, 1987) and it probably mediates some aspects of endotoxic shock (Beutler *et al.*, 1985; Waage *et al.*, 1987). TNF is a candidate for the biological treatment of cancer in view of its well established activity against experimental cancer. We administered rhTNF to cancer patients in order to find the maximum tolerated dose, study its pharmacokinetics and make an initial evaluation of its anticancer effect. We were also able to make some observations which may contribute to our understanding of the biology of TNF in man.

### Patients, materials and methods

### *Study design*

New biological materials present special difficulties for early pharmacological studies. Toxicity is unpredictable and major species differences between tested species and man are possible. We adopted a low starting dose and cautious dose escalation.

Human recombinant TNF is lethal to 10% of mice at a dose of $9 \times 10^6$ units m$^{-2}$ i.v. The conventional starting dose for studies with a new anti-cancer drug with this toxicity in mice would therefore have been $9 \times 10^5$ units m$^{-2}$ (Von Hoff *et al.*, 1984). We elected to begin treatment at a dose of $9 \times 10^3$ units m$^{-2}$ (3 patients). The study design allowed a patient to receive 2 treatments at the initial dose separated by 2 weeks and then one dose escalation for the third dose, 2 weeks later (Table I). Dose levels for escalation were: $9 \times 10^4$ units m$^{-2}$ (6pts), $3 \times 10^5$ units m$^{-2}$ (6pts), $6 \times 10^5$ units m$^{-2}$ (5pts), $9 \times 10^5$ units m$^{-2}$ (7pts), $1.2 \times 10^6$ units m$^{-2}$ (2pts).

Between 3 and 5 patients began treatment at each dose

**Table I** Study design and patient numbers

| | *No. pts* | *First dose* | *Second dose* | *Third dose* |
|---|---|---|---|---|
| Group 1 | 3 | $9 \times 10^3$ | $9 \times 10^3$ | $9 \times 10^4$ |
| Group 2 | 3 | $9 \times 10^4$ | $9 \times 10^4$ | $3 \times 10^5$ (1pt withdrawn) |
| Group 3 | 4 | $3 \times 10^5$ | $3 \times 10^5$ | $6 \times 10^5$ (2pts withdrawn) |
| Group 4 | 3 | $6 \times 10^5$ | $6 \times 10^5$ | $9 \times 10^5$ (1pt received $6 \times 10^5$) |
| Group 5 | 5 | $9 \times 10^5$ | $9 \times 10^5$ (2pts withdrawn) | $1.2 \times 10^5$ (3pts withdrawn) |

Correspondence: P. Selby.
Received 20 July 1987; and in revised form, 1 October 1987.

level. When 3 patients had adequately tolerated the initial dose (no more than World Health Organisation grade II toxicity; WHO, 1979) one escalation in starting dose was made for the next patient so that most patients received two different doses. In 5 patients improvement in their disease was suspected and treatment was continued as long as was clinically indicated.

*Drug administration and observation*

Patients were nursed in an intensive care unit with continuous monitoring of pulse, electrocardiograph and blood pressure for 24 h. Full blood counts, biochemical tests and urinalysis were repeated 4 h after drug administration. Subsequently, clinical observations, full blood counts, urinalysis and biochemistry were repeated daily for 7 days in hospital. As the study progressed, patients were in part observed daily as outpatients during the 7 days.

*Patients*

Eighteen patients were treated in the study. Inclusion criteria were a diagnosis of advanced cancer for which no conventional treatment existed, informed consent, 18 yrs of age or over, performance status 2 or less (WHO, 1979), normal renal, hepatic and respiratory function and a normal full blood count. In fact, one patient age 17 yrs, one with a marginally raised serum creatinine (111 $\mu$mol $l^{-1}$) and one with thrombocytopenia were included. Diagnoses were non-Hodgkin lymphoma (5), Hodgkin's disease (1) metastatic malignant melanoma (6), liposarcoma (1), gastrointestinal cancer (3), lung cancer (2). Eleven were male and 7 female. Mean age was 41 years (range 17–57); WHO performance status was 0 in 4 patients, 1 in 4 patients, and 2 in 10 patients. All had previously received conventional cytotoxic chemotherapy without control of their disease.

*TNF*

Human recombinant TNF was provided by the Asahi Chemical Industry Company prepared as previously described (Shirai *et al.*, 1985). It is highly purified and there is no evidence of residual bacterial endotoxin. Freeze-dried TNF was stored at +4°C and reconstituted in sterile water and then further diluted in 100 mls saline, before administration over 1 h i.v. The specific activity of the preparation was $2.2 \times 10^6$ units $mg^{-1}$. A unit is arbitrarily defined as the reciprocal of the dilution required to produce 50% cell survival *in-vitro* for a sensitive murine cell line (L-M cells). Different laboratories use different cell strains, and doses in *units* may *NOT* be comparable to ours.

*TNF assay*

TNF levels in plasma were measured with a two-site enzyme-linked immunosorbant assay (ELISA) using two different anti TNF monoclonal antibodies. Briefly, one monoclonal antibody was immobilised in 96-well microplates. The plates were washed and test and standard samples were reacted with this antibody. After further washing, a second monoclonal antibody conjugated to horseradish peroxidase was added. This was washed and then substrate added. The absorbance of the product was measured to estimate TNF concentration. The assay will detect TNF above a concentration of 0.1 units $m^{-1}$ (45 pg $ml^{-1}$).

*C-reactive protein (CRP)*

Serum CRP was measured in 7 patients as an indication of an acute phase protein response. Sera were stored at −20°C and CRP assayed using a standard radial immuno-diffusion technique using antisera and standards obtained from Behringwerke (AG), Marburg, West Germany. This assay detects CRP at 10 mg $l^{-1}$ and levels above 10 mg $l^{-1}$ are used by its developers as evidence of an acute phase response (Cooper & Stone, 1979).

**Results**

*Toxicity*

The first 6 patients treated all experienced acute febrile reactions with moderate or severe rigors which were not dose-related in severity or incidence. These reactions represent the unmodified acute biological response to TNF and are described in some detail. The rigors developed 20 min after the beginning of the infusion (median, range 15–50) and lasted 20 min (median, range 15–30). The infusions were stopped and restarted when the rigors were complete and no further acute reactions occurred. Fever was noted 1 h after the beginning of the infusion of TNF, rose to a maximum at 1.6 h and median maximum fever was 38.2°C (range 37.4–39°C). It subsided over 1–4 h and was monophasic. Tachycardia (median 127 $min^{-1}$) and minor hypertension (median diastolic increase 10 mm Hg) were associated with the reactions. Maximum tachycardia occurred in the first 3 h (range 0.25–3 h) and preceded the onset of hypotension (see below).

A range of manoeuvres were tried to minimise the acute reactions using corticosteroids, sedatives, non-steroidal anti-inflammatory drugs and antihistamines. No treatment seemed to modify established reactions. Hydrocortisone and chlorpheniramine pre-treatment were ineffective. Pre-treatment with i.v. methyl prednisolone (250–500 mg) 2 h before TNF and oral indomethacin (50 mg) dramatically reduced the rigors, fevers, tachycardia and hypertension. Sixteen of 23 patients who received this prophylaxis experienced no reaction to TNF at all and in the others they were only mild. The controllable acute febrile reactions were not considered a dose-limiting toxicity. However, as higher doses of rhTNF were given several other toxicities were seen:

*Hypotension* Mild hypotension was seen at doses of TNF less than $9 \times 10^5$ u $m^{-2}$ with minimum b.p. 105/60 (median) and no systolic pressure <80 mm Hg and/or diastolic <55 mm Hg. However, at a dose of $9 \times 10^5$ u $m^{-2}$, 3/7 patients developed hypotension with diastolic <50 mm Hg and/or systolic <80 mm Hg and in one case this was severe 50/35 (from baseline 120/60) and life threatening requiring i.v. fluids and a dopamine infusion. A fall in blood pressure (40 mm and 20 mm of Hg systolic respectively) occurred in both patients treated at $1.2 \times 10^6$ u $m^{-2}$. Hypotension was most severe 6–12 h after TNF and occurred despite the use of methyl prednisolone pre-treatment. One patient developed minimum b.p. of 90/40 after $9 \times 10^5$ u $m^{-2}$ but only 115/55 after $6 \times 10^5$ u $m^{-2}$ which also supports the dose relationship of the hypotension.

*Abnormal hepatic enzymes* No substantial abnormalities in LFTs were seen at or below a dose of $3 \times 10^5$ u $m^{-2}$. Two of 5 patients who received $6 \times 10^5$ u $m^{-2}$ and 3 of 7 patients who received $9 \times 10^5$ u $m^{-2}$ developed transient abnormalities of more than WHO grade II (WHO, 1979) severity in one or more hepatic enzyme usually alkaline phosphatase. All returned to pre-treatment levels before the next dose of TNF. No cummulative abnormalities were noted with repeated treatments.

*Changes in white cell count* No changes were seen at doses of $9 \times 10^3$ u $m^{-2}$. Between $9 \times 10^4$ and $6 \times 10^5$ units $m^{-2}$ most patients developed neutrophil leucocytosis of $16–30 \times 10^9$ $l^{-1}$ at 24 h returning to normal over a further 24 h. At a dose of $9 \times 10^5$ u $m^{-2}$ profound leucopenia developed in 4 of 7 patients and was less than $1 \times 10^9$ leucocytes $l^{-1}$ in 3 patients. This was very short-lived and white cell counts returned to normal levels at 24 h.

*Changes in renal function* Elevated creatinine levels to WHO toxic levels I occurred in 4pts at doses of $9 \times 10^5$ and $1.2 \times 10^6$ and recovered over 2–3 days. Traces of proteinuria developed on day 2–3 after TNF in 6pts at various doses, and resolved over 1–2 days. No evidence of weight gain or fluid retention was seen.

The toxicity observations suggest that a dose of $9\times10^5$ u m$^{-2}$ (400 µg) of rhTNF or greater is associated with a considerable risk of clinically significant hypotension, hepatic abnormalities and transient leucopenia and should be regarded as the maximum tolerated dose in man. Each of these toxicities contributed to the dose limitation.

*Anticancer effect*

Evidence of possible anticancer effect was seen in three patients.

1. A 44 year old man with mediastinal diffuse large cell lymphoma had failed to respond to extensive chemotherapy and radiotherapy and had extensive solid pleural disease seen on X-ray and ultrasound. He received rhTNF $9\times10^4$ u m$^{-2}$ twice and then $3\times10^5$ u m$^{-2}$ once. His disease visible on CXR regressed but he died of pneumonia and radiation pneumonitis 1 month later. Concomitant medication included high doses of methylprednisolone and, although he had previously failed to respond to large doses of corticosteroids, this cannot be excluded as a factor in the regression of his disease.
2. A 29 year old man with extensive nodular sclerosing Hodgkin's disease resistant to conventional treatment received rhTNF $3\times10^5$ u m$^{-2}$ twice followed by $6\times10^5$ u m$^{-2}$ on six occasions as an extension of the study. His severe symptoms resolved and there was some clearing of lung disease on X-ray. When rhTNF was stopped, the symptoms recurred after 2 months and then failed to respond to TNF.
3. A 37 year old man with splenic and bone marrow non-Hodgkin lymphoma resistant to all conventional therapy, had severe bone marrow failure with life-threatening thrombocytopenia ($11\times10^9$ l$^{-1}$). A marrow aspirate contained 100% lymphoma cells. He received rhTNF $9\times10^5$ u m$^{-2}$ twice, $1.2\times10^6$ u m$^{-2}$ once and then prolonged treatment with $6\times10^5$ u m$^{-2}$ two weekly. His spleen regressed from 10 cm below costal margin in its long axis to 4 cm and his platelet count has risen to $64\times10^9$ l$^{-1}$ and is presently sustained after 3 months without treatment. His marrow contains 20% lymphoma cells. He has been receiving low dose prednisolone without apparent benefit for 6 months before TNF therapy was given and he continued the steroids during his TNF treatment.

Although symptomatic improvement occurred in several other patients, no objective evidence of response was seen.

*Acute phase proteins*

Serum C-reactive protein concentration was measured before and 24 h after rhTNF (6–$9\times10^5$ m$^{-2}$) in 7 patients. In 3 of these levels were taken for 2 treatments and in one patient for 3 treatments. Results are shown in Figure 1. Substantial

**Figure 1** C-reactive protein levels before and after rhTNF.

**Figure 2** Plasma TNF after intravenous treatment in two patients. (a) exponential decay at two dose levels but with more rapid clearance at lower dose; (b) exponential decay at the lower dose but a convex upward curve at higher dose indicating slower clearance at high concentrations.

increases were seen in 6 patients and in each case was seen with each treatment although the amount of the increase varied between patients and occasions. Increased CRP was seen in patients who did not have rigors and was not prevented by methylprednisolone treatment. One patient (tested on one occasion only) showed no rise after $6 \times 10^5 \mathrm{u\,m}^{-2}$ rhTNF despite a moderately severe rigor.

*Pharmacokinetics*

Pharmacokinetics of rhTNF were studied in 17 patients who consented to blood sampling within the study and repeated studies were possible in 11 patients. Samples were taken through indwelling venous lines before and immediately after rhTNF infusions and then at 15, 30, 45, 60, 90, 120, 180 and 240 min.

The results are shown in Figures 2(a, b) and 3 and in Table II. No rhTNF was detected in the urine.

In 8 patients, the concentrations of rhTNF fell exponentially on at least one occasion (as shown for 2 patients in Figure 2) and median half life was 17 min (range 8–61 min). In the remaining 9 patients, the plasma decay curves were not exponential and half lives were not calculated. In 3 of these 9 patients the data were scattered and no satisfactory shape to the plasma concentration curves could be discerned. In 6 patients the curves (log concentration *vs.* time) were convex upwards (as illustrated for one patient in Figure 2b) with less steep slopes at higher concentrations. In two patients who showed exponential fall in plasma concentrations on one occasion, convex-upwards curves were seen on other occasions (Figure 2).

Peak plasma concentrations were read directly from the curves and areas under the plasma concentration-times curves were estimated by the trapezoid rule and clearance calculated from this (Table II). Peak concentration and AUC increased with dosage. However, clearance of TNF fell with increasing dose (Figure 3) demonstrating non-linear pharmacokinetics.

**Figure 3** Calculated clearance of rhTNF at different dose levels in 17 patients (—mean clearances).

There was considerable variation between patients and somewhat less between studies in the same patient. These may be compared at a dosage of $6 \times 10^5 \mathrm{u\,m}^{-2}$ where most replication of measurements was possible and the coefficient of variation (standard deviation/mean %) of AUC between patients was 45% whereas the average coefficient of variation of results within one patient was 27%. The variations between patients could not be explained by sex, age, diagnosis, organ dysfunction, apparent body fat or previous TNF administration in this study.

*TNF levels in cancer patients*

Endogenous plasma TNF levels were measured in 72 patients untreated with TNF who had malignancy including 51 with lymphoma and 10 with severe cachexia. Samples were taken, separated and stored at −20°C for several weeks before being assayed using the two-antibody method. TNF levels were below the lowest reliable measurements possible with this assay (0.1 u ml$^{-1}$, 45 pg ml$^{-1}$) at the time of measurement in these cryopreserved samples.

### Discussion

This study allows some conclusions to be drawn about tumour necrosis factor in man. Firstly we can conclude that the drug can be administered safely to patients in doses up to $9 \times 10^5$ units m$^{-2}$ but that at that dose a substantial proportion of patients develop significant toxicities including transient leucopenia, hypotension, abnormalities in liver functions tests and mild renal impairment. The maximum frequency of administration is not yet known. The febrile reactions which were seen at all doses can be prevented by steroids and indomethacin. However, such prophylaxis may not be desirable because its influence on possible therapeutic benefits is unknown. Some preliminary observations suggest it may reduce the anticancer effect in experimental systems (unpublished). It is notable that the dose at which significant toxicity was seen would have been a conventional first dose for a new cytotoxic drug supporting our belief that additional caution is required in early clinical studies of new biological treatments.

The lower clearance of TNF seen at higher dosages together with the convex-upward log concentration-time curves seen in some patients show that TNF has non-linear pharmacokinetics. It appears that a clearance mechanism is saturated by high concentrations but the exact explanation for the non-linear pharmacokinetics and its biological significance is unclear. The concentrations of TNF achieved in the serum are higher than those required to kill some human cells in tissue culture but are somewhat lower than those associated with regression of most murine experimental tumours (Matthews & Watkins 1978; Matthews, 1978 & unpublished observations).

We are encouraged by the evidence for some regression of tumours in three patients and further exploration of this material as a new biological therapy for cancer, seems justified. The apparent effects on lymphomas may be related to the known cytotoxic effects of TNF on normal mouse

**Table II** Pharmacokinetics of rhTNF

| *Dose* u m$^{-2}$ | *Pts* | *Studies* | *Max conc (s.d.)* u ml$^{-1}$ | $t_{\frac{1}{2}}$ *(s.d.) min* | *AUC (s.d.)* u ml$^{-1}$ *min* | *VOD (s.d.) litres* | *Clearance (s.d.)* ml min$^{-1}$ |
|---|---|---|---|---|---|---|---|
| $9 \times 10^3$ | 3 | 3 | 2.1(2.4) | – | – | – | – |
| $9 \times 10^4$ | 4 | 4 | 4.5(2.7) | 10.4(4.6) | 364(134) | 7.3 (5.0) | 457(145) |
| $3 \times 10^5$ | 6 | 6 | 39.4(22.3) | 15.3(4.3) | 2326(1794) | 4.8 (2) | 232(116) |
| $6 \times 10^5$ | 5 | 12 | 120(50) | 21.4(5.4) | 9861(4624) | 3.6 (1.7) | 152(104) |
| $9 \times 10^5$ | 4 | 8 | 217(101) | 26.5(11.7) | 24,942(11117) | 2.59(0.85) | 73(47) |
| $1.2 \times 10^6$ | 2 | 2 | 583 | 41.5 | 45,712 | 2.6 | 49.5 |

lymphoid cells (Playfair *et al.*, 1982). However, it must be emphasised strongly that it is difficult to draw conclusions about a consistent anticancer effect in man from these studies and we do not claim on the basis of the available evidence that this drug has yet been shown to be significant new treatment.

The occurrence of fevers and rigors after low doses of TNF suggests that it is an endogenous pyrogen. In rabbits TNF is pyrogenic directly as well as by the release of interleukin 1 which results in a biphasic fever (Dinarello *et al.*, 1986). There is no evidence of biphasic fever in man. Apart from the production of fever, the toxicity pattern suggests that TNF may mediate hypotension, abnormal liver function tests and renal failure in some infectious diseases. This suggestion is supported by the observation in experimental animals that anti TNF antibodies reduce fatalities resulting from experimental infections (Beutler *et al.*, 1985) and the association of high TNF levels with fatal meningococcal infection (Waage *et al.*, 1987). It is possible that the production of TNF may also have a role in the defence against infections. The activity of the material against parasites *in vitro* and the observation that serum TNF levels are elevated in some parasitic infections (Scuderi *et al.*, 1986) would support this suggestion.

We have found no evidence of elevation of serum TNF levels in any patients with malignant disease and this agrees with some other results (Scuderi *et al.*, 1986). However, we are using a double-antibody method to measure intact TNF and have only looked at cryopreserved samples. It is possible that the production of TNF from peripheral blood monocytes is increased in cancer patients (Aderka *et al.*, 1985) and we are exploring this possibility.

A role for TNF in mediation of the acute phase protein response in animals has already been proposed (Dinarello, 1986). Moreover, there is evidence that TNF may regulate the expression of hepatic acute phase protein genes *in vitro* (Perlmutter *et al.*, 1986). The data presented here suggests that TNF may indeed mediate the production of some acute phase proteins in man.

TNF appears to be of considerable biological importance although its role as a cancer therapy will only become clear when further studies have evaluated treatment at doses which we have found to be tolerated and more frequent administration. The combination of TNF with other biological agents such as gamma interferon has been shown in experimental systems to be strongly synergistic (Aggarwal *et al.*, 1985; Fransen *et al.*, 1986; Balkwill *et al.*, 1987) and this will justify clinical evaluation of the combination. Combinations of biological agents with conventional cytotoxic drugs appear also to be effective in experimental systems and deserve consideration (unpublished data).

### References

ADERKA, D., FISHER, S., LERO, Y., HOLTMAN, H., HANN, T. & WALLACH, D. (1985). Cachectin/tumour necrosis factor production by cancer patients. *Lancet*, **ii**, 1190.

AGGARWAL, B.B., FESSALU, T.E. & HASS, P.E. (1985). Characterisation of receptors for human tumour necrosis factor and their regulation by gamma-interferon. *Nature*, **318**, 665.

BALKWILL, F.R., WARD, B.G. & FIERS, W. (1987). Anti tumour effects of TNF on human tumour xenografts in nude mice. Ciba Symposium on TNF and related cytotoxins. (In press).

BERTOLINI, D.R., NEDWIN, G.E., BRINGMAN, T.S., SMITH, D.D. & MUNDY, G.R. (1986). Stimulation of bone resorption and inhibition of bone formation *in vitro* by human tumour necrosis factors. *Nature*, **319**, 516.

BEUTLER, B. & CERAMI, A. (1987). Cachectin: More than just a tumour necrosis factor. *New Engl. J. Med.*, **316**, 379.

BEUTLER, B., MILSARK, I.W. & CERAMI, A.C. (1985). Passive immunisation against cachectin/tumour necrosis factor protects mice from lethal effects of endotoxins. *Science*, **229**, 869.

CARSWELL, E.A., OLD, L.J., KASSEL, R.L., GREEN, S., FIORE, N. & WILLIAMSON, B. (1975). An endotoxin-induced serum factor that causes necrosis of tumours. *Proc. Natl Acad. Sci. USA.*, **72**, 3666.

COOPER, E.H. & STONE, J. (1979). Acute phase reactant proteins in cancer. *Adv. Cancer Res.*, **30**, 1.

DINARELLO, C.A. (1986). Interleukin I: Amino acid sequences, multiple biological activities and comparisons with tumour necrosis factor (cachectin). *Year in Immunology*, p. 68.

DINARELLO, C.A., CANNON, J.G., WOLFF, S.M. & 6 others (1986). Tumour necrosis factor (cachectin) is an endogenous pyrogen and induces production of interleukin I. *J. Exp. Med.*, **163**, 1433.

FRANSEN, L., VAN DER HAYDEN, J., RUYSSCHAERT, R. & FIERS, W. (1986). Recombinant tumour necrosis factor: Its effect and its synergism with gamma interferon on a variety of normal and transformed human cell lines. *Eur. J. Cancer Clin. Oncol.*, **22**, 419.

KULL, F.C., JACOBS, S. & CUATRECASAS, P. (1985). Cellular receptor for labelled human tumour necrosis factor. *Proc. Natl Acad. Sci. USA.*, **82**, 5756.

MARMENOUT, A., FRANSEN, L., TAVERNIER, J. & 6 others (1985). Molecular cloning and expression of human tumour necrosis factor and comparison with mouse tumour necrosis factor. *Eur. J. Biochem.*, **152**, 516.

MATTHEWS, N. & WATKINS, J.F. (1978). Tumour necrosis factor from the rabbit. 1. Mode of action, specificity and physicochemical properties. *Br. J. Cancer*, **38**, 302.

MATTHEWS, N. (1978). Tumour necrosis factor from the rabbit. II. Production by monocytes. *Br. J. Cancer*, **38**, 310.

MESTAN, J., DIGEL, W., MITTNACHT, S. & 5 others (1986). Antiviral effects of recombinant tumour necrosis factor *in vitro*. *Nature*, **323**, 816.

NEDWIN, G.E., NAYLOR, S.L., SAKAGUCHI, A.Y. & 5 others (1985). Human lymphotoxin and tumour necrosis factor genes: Structure, homology and chromosomal localisation. *Nucleic Acids Res.*, **13**, 6361.

OLD, L.J. (1985). Tumour necrosis factor (TNF). *Science*, **230**, 630.

PENNICA, D., NEDWIN, G.E., HAYFLICK, J.S. & 6 others (1984). Human tumour necrosis factor: Precursor structure, expression and homology to lymphocytotoxin. *Nature*, **312**, 724.

PERLMUTTER, D.H., DINARELLO, C.A., PISMAL, I. & COLTEN, H.R. (1986). Interleukin I and cachectin/tumour necrosis factor regulate hepatic acute phase genes. In *Protides of the Biological Fluids*, Peeters, H. (ed) p. 235.

PLAYFAIR, J.H.L., DE SOUZA, J.B. & TAVERNE, J. (1982). Endotoxin induced tumour necrosis serum kills a subpopulation of normal lymphocytes *in vitro*. *Clin. Exp. Immunol.*, **47**, 753.

RUBIN, B.Y., ANDERSON, S.L., SULLIVAN, S.A., WILLIAMSON, B.D., CARSWELL, E.A. & OLD, L.J. (1985). High affinity binding of labelled human tumour necrosis factor to specific cell surface receptors. *J. Exp. Med.*, **162**, 1099.

SATO, N., GOTO, T., HARANAKA, K. & 4 others (1986). Actions of tumour necrosis factor on cultured vascular endothelial cells: Morphological modulation, growth inhibition and cytotoxicity. *J. Natl Cancer Inst.*, **76**, 1113.

SCUDERI, P., STERLING, K.E., LAN, K.S. & 6 others (1986). Raised serum levels of tumour necrosis factor in parasitic infections. *Lancet*, **ii**, 1364.

SHIRAI, T., YAMAGUCHI, H., ITO, H., TODD, C.W. & WALLACE, R.B. (1985). Cloning and expression in E coli of the gene for human tumour necrosis factor. *Nature*, **313**, 803.

TAVERNE, J., DOCKRELL, H.M. & PLAYFAIR, J.H.L. (1981). Endotoxin induced serum factor kills malarial parasites *in vitro*. *Infect. Immun.*, **33**, 83.

TAVERNE, J., MATTHEWS, N., DEPLEDGE, P. & PLAYFAIR, J.H.L. (1984). Malarial parasites and tumour cells are killed by the same component of tumour necrosis serum. *Clin. Exp. Immunol.*, **57**, 293.

VILCEK, J., PALOMBELLA, V.J., HENRIKSON-DE STEFANO, D. & 4 others (1986). Fibroblast growth enhancing activity of tumour necrosis factor and its relationship to other polypeptide growth factors. *J. Exp. Med.*, **163**, 632.

von HOFF, D.D., KUHN, J. & CLARK, G.M. (1984). Design and conduct of phase I trials. In *Cancer Clinical Trials. Methods and Practice*, Buyse, M.E. *et al.* (eds) p. 210. Oxford University Press: Oxford.

WAAGE, A., HALSTENSEN, A. & ESPEVIK, T. (1987). Association between tumour necrosis/factor in serum and fatal outcome in patients with meningococcal disease. *Lancet*, **ii**, 355.

WHO (1979). *Handbook for reporting results of Cancer Treatment*. Offset publication 48. WHO, Geneva.

WANG, A.M., CREASEY, A.A., LADNER, M.B. & 5 others (1985). Molecular cloning of the complementary DNA for human tumour necrosis factor. *Science*, **288**, 149.

WONG, G.H.W. & GOEDDEL, D.V. (1986). Tumour necrosis factors can inhibit virus replication and synergize with interferons. *Nature*, **323**, 819.